# Development of Wood Apple Shell (*Feronia acidissima*) Powder Biosorbent and Its Application for the Removal of Cd(II) from Aqueous Solution

**DOI:** 10.1155/2014/154809

**Published:** 2014-04-07

**Authors:** Ch. Suresh, D. Harikisore Kumar Reddy, Yapati Harinath, B. Ramesh Naik, K. Seshaiah, Annareddy V. Ramana Reddy

**Affiliations:** ^1^Inorganic and Analytical Chemistry Division, Department of Chemistry, Sri Venkateswara University, Tirupati 517 502, India; ^2^Analytical Chemistry Division, Bhabha Atomic Research Centre, Trombay, Mumbai 400 085, India

## Abstract

A biosorbent was prepared by using wood apple shell (WAS) powder and studied its application for the removal of Cd(II) from aqueous solution by a batch method. The biosorbent was characterized by infrared spectroscopy, X-ray diffraction, scanning electron microscopy, and elemental analysis. WAS is principally made up of lignin and cellulose, containing functional groups such as alcoholic, ketonic, and carboxylic groups which can be involved in complexation reactions with Cd(II). The effect of experimental parameters like initial pH, contact time, metal ion concentration, and sorbent dose on adsorption was investigated. The optimum pH for biosorption of Cd(II) onto WAS was found to be pH 5.0 and the quantitative removal of Cd(II) ions was achieved in 30 min. The kinetic study showed that the biosorption process followed the pseudo-second-order rate. Experimental data were analyzed by Langmuir, Freundlich, and Dubinin-Radushkevich isotherm models. Desorption studies were carried out using HCl solution.

## 1. Introduction

Water pollution by heavy metals has become a serious global concern because metals are nonbiodegradable and are toxic to human beings [1, 2]. Among the heavy metal ions cadmium is one of the extremely toxic and carcinogenic metal ion due to its solubility and mobility in aqueous solutions [3]. Even at low concentrations cadmium is highly toxic to living organisms [4, 5]. In case of humans cadmium toxicity can cause renal dysfunction, hypertension, hepatic injury, lung damage, and teratogenic effects [6]. In 1950, due to usage of municipal sewage sludge as a fertilizer in rice cultivation, cadmium toxicity in humans was reported in Japan [7]. Cd(II) enters into water bodies through various industrial discharges like smelting, metal plating, cadmium-nickel batteries, and alloy industries, and also it enters through sewage sludge [8]. In view of its toxicity, cadmium is listed by the US Environmental Protection Agency (USAEPA) as one of the 129 priority pollutants [9] and set 0.01 mg L^−1^ as safety level in drinking water [10].

Because of its toxicity, different treatment methods and techniques have been developed for the removal of Cd(II) from aqueous solution. The most commonly employed methodologies for the treatment of cadmium containing wastewater include chemical precipitation, ion exchange, and membrane separation. Although the effectiveness of these methods has been proved, these methods involve high capital cost with recurring expenses which necessitated for the development of effective and economical methods for the removal of cadmium from water and wastewater. In recent years there is a growing interest in developing eco-friendly biomaterials for the removal of metal ions from aqueous solutions [11–14]. Treatment of metal contaminated wastewater with biomaterial has emerged as an economical technology in waste water treatment because these materials can be generated from agricultural and industrial waste by-products, for example, WAS.

The literature survey revealed that the wood apple (*Feronia acidissima*) contains polar functional groups such as alcoholic, carboxylic, and ether [25]. Further, Wood Apple is a native and common fruit cultivated in the dry plains of India, Sri Lanka, Thailand, and Cambodia. Wood apple tree parts such as bark, leaves, and fruits have medicinal importance. Fruit is edible after removing the shell, and the shell is discarded as agricultural waste. Disposal of large amounts of shells directly in the soil may contaminate the environment in an uncontrolled way because the decomposition of this waste material leads to the generation of various chemical compounds and microorganisms. Hence, preparation of biosorbent from wood apple shell powder provides economic and environmental advantages for developing countries such as India.

In present investigation, application of wood apple shell (WAS) powder as biosorbent for the removal of Cd(II) from aqueous solutions has been systematically investigated. The biosorbent was characterized by various analytical techniques. Various operating parameters such as effect of pH, adsorbent dose, initial metal ion concentrations and contact time on biosorption efficiency were also studied. Equilibrium biosorption data was analyzed by various isotherm models such as Langmuir, Freundlich, and Dubinin-Radushkevich. Results have been discussed in the light of its utility as biosorbent and the possible mechanism involved in the sorption.

## 2. Experimental

### 2.1. Biosorbent Preparation

Wood Apple shells were collected in local fields of Doruvupadu village in Nellore district (Andhra Pradesh, India). The shells were made into small pieces, washed several times in deionized water, dried under sunlight, and grounded in a steel mill to get fine powder. The fine powder was washed to remove soluble material with deionized water and dried at 70°C temperature in hot air oven for about 3 hours. The final product was named as WAS (wood apple shell).

### 2.2. Chemicals and Equipment

All chemicals used in this study were of analytical reagent grade. Deionized double distilled water (DDW) was used throughout the experimental studies. Stock cadmium solution (1 mg mL^−1^) was prepared by dissolving appropriate quantity of Cd (NO_3_)_2_·4H_2_O in 1% HNO_3_. Working standards were prepared by progressive dilution of stock cadmium solution using DDW. ACS reagent grade HCl, NaOH, and buffer solutions (E. Merk) were used to adjust the solution pH. An Elico (LI-129) pH meter was used for pH measurements. The pH meter was calibrated using standard buffer solutions of pH 4.0 and 9.2. FT-IR spectrometer (Thermo-Nicolet FT-IR, Nicolet IR-200, USA) was used for the IR spectral studies (4000–400 cm^−1^) of biosorbent. Vario EL, Elementar, Germany, was used for elemental analysis of the WAS. The cadmium concentration in the samples was determined by atomic absorption spectrophotometer (AAS, Shimadzu AA-6300) with cadmium Hallow Cathode Lamp. Samples for X-ray diffraction measurements were prepared by grounding the adsorbent with a small volume of methanol in an agate mortar. The mixture was smear-mounted onto the zero-background quartz window specimen holder and allowed to air dry. Wide angle X-ray diffraction (WAXD) patterns of samples were recorded on an X-ray diffractometer (XRD 6000, Shimadzu), using K*α* radiation (= 1.5406 Å) at 40 kV voltage and 30 mA of current.

### 2.3. Batch Biosorption Process

A fixed amount (200 mg) of WAS biomass was placed in 250 mL stoppered flask and 25 mL of aqueous cadmium solution was added. The sealed bottle was placed in mechanical shaking incubator and shaken for 3 hours at a speed of 300 rpm and temperature of 25°C. The contents of the flask were filtered through a Whatman filter paper and the concentration of Cd in the filtrate was determined by flame AAS method. The effect of pH on Cd(II) ion biosorption was studied by equilibrating the WAS in solutions with initial pH in the range of 2.0–8.0 by adjusting the pH with diluted HCl or NH_3_ and buffer solution. The effect of contact time on Cd(II) removal by WAS was studied by varying the contact time from 5 to 70 min. For the adsorption isotherm studies, the initial metal ion concentration was varied in the range of 10–1000 mg L^−1^ by keeping the WAS dose constant at 200 mg. The amount of metal ion sorbed (*Q*_*e*_) was computed by the following equation:

(1) $$\begin{array}{c} Q_{e}=\frac{v}{m}\left(C_{0}-C_{e}\right), \end{array}$$

where *C*_0_ and *C*_*e*_ are the initial and equilibrium Cd(II) concentrations, respectively, whereas *v* and *m* are solution volume and mass of biosorbent, respectively. Blank test was performed in the same experimental conditions without biosorbent.

### 2.4. Desorption Studies

The desorption of Cd(II) from metal loaded WAS was carried out by using 0.1 M HCl. After determination of metal content in the final solutions, the biosorbent was washed with excess of acid solution and distilled water in order to reuse the WAS.

## 3. Results and Discussion

### 3.1. Characterization of the Biosorbent

Elemental analysis results showed that WAS is composed of 47.66 ± 2.35% carbon, 5.89 ± 0.03% hydrogen, 0.10 ± 0.01% nitrogen, and 45.58 ± 1.45% oxygen. Other characteristics of biosorbent are given in Table 1.

#### 3.1.1. FT-IR Spectra

**Figure 1:**
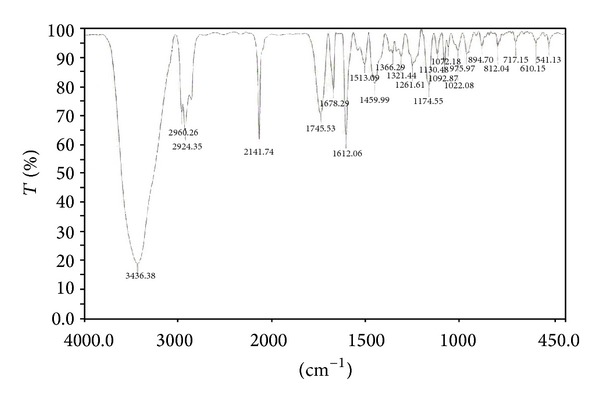
FTIR spectra of WAS.

To investigate the functional groups present on the surface of WAS and the groups involved in sorption of Cd(II) onto WAS, FT-IR study was carried out and the spectra were shown in Figure 1. The FTIR spectrum of WAS showed a number of absorption peaks, reflecting the presence of complex functional groups on the surface of the biosorbent. A peak at 3436 cm^−1^ is due to the stretching of the N–H of amino groups and indicative of bonded hydroxyl group [26]. The strong absorption peak at 2924 cm^−1^ could be assigned to –CH stretching vibrations of –CH_3_ and –CH_2_ functional groups. The peak at 1612 cm^−1^ indicates the fingerprint region of CO, C–O, and O–H groups [27]. The region between 1520 and 1000 cm^−1^ is the finger print of OH, and C–H bending vibration and C–O stretching vibration. The peaks at 1459, 1366, and 1321 cm^−1^ could be attributed to the presence of C–O stretching. The intense band at 1072 cm^−1^ can be assigned to the C–O of alcohols and carboxylic acids. The FT-IR spectral studies showed that WAS is principally made up of lignin and cellulose, containing functional groups such as alcoholic, ketonic, and carboxylic groups. These groups can be involved in complexation reactions with Cd(II) and WAS could be viewed as a natural ion-exchange material that primarily contains weak acidic and basic groups on the surface.

#### 3.1.2. X-Ray Diffraction (XRD)

**Figure 2:**
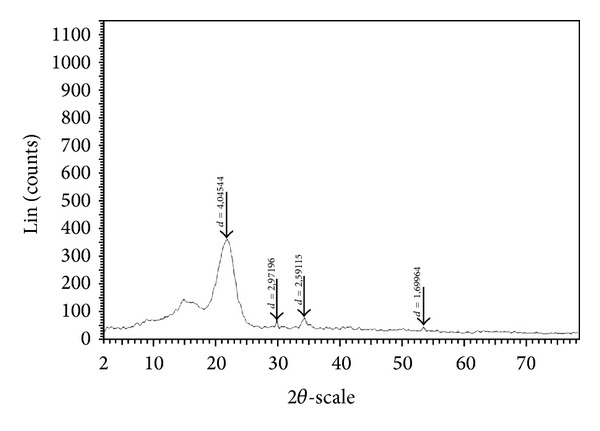
Powder XRD of WAS.

The crystal phases of WAS were determined by X-ray powder diffraction (Figure 2). The X-ray diffraction spectrum of WAS biosorbent indicates amorphous regions and peaks at 22°, 30°, 34°, and 54°. This is comparable with natural fibers which exhibit amorphous regions with the presence of some definite X-ray diffraction lines. It is well known that cellulose from widely different sources such as cotton, ramie, and wood contains amorphous regions [28, 29]. The XRD study revealed the presence of amorphous regions, which is favorable for biosorption of metal ion onto WAS.

#### 3.1.3. Scanning Electron Microscopy

**Figure 3:**
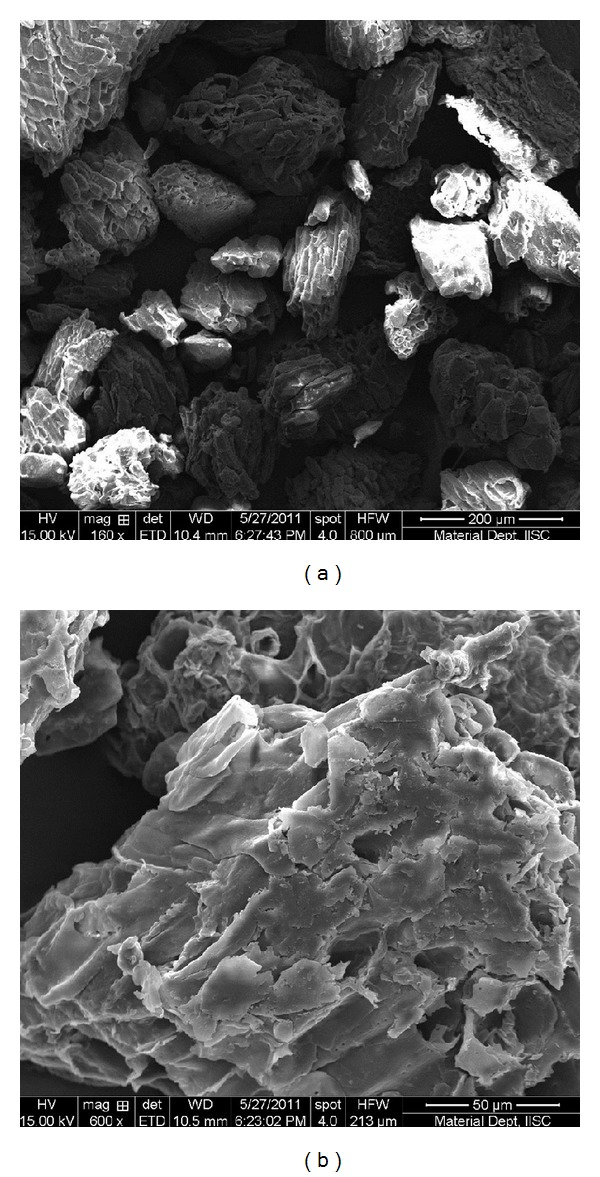
SEM images of WAS at two different magnifications.

To characterize the textural properties of wood apple shell, the biosorbent morphology was analyzed by SEM. SEM image of WAS (Figure 3) shows rough, uneven, and heterogeneous surface with porous structure, which indicates the availability of high surface area for biosorption of metal ion.

### 3.2. Effect of pH on Removal of Cadmium

**Figure 4:**
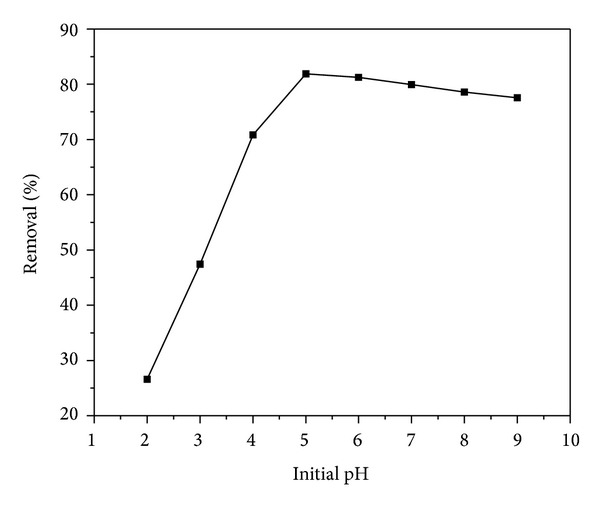
Effect of initial pH on the removal of Cd(II) on WAS.

Among all other parameters pH of the solution has been found to be the most important in influencing adsorbate and adsorbent interactions in aqueous solutions [30, 31]. In order to study the effect of pH on sorption of Cd(II) onto WAS, batch equilibrium studies were conducted at different initial pH in the range of 2.0–9.0 (Figure 4). It can be noticed that the removal of Cd(II) increased with increasing pH of aqueous solution and reached maximum sorption at pH 5.0. Above the pH 5.0 a marginal decrease in the biosorption of metal ion was observed. The removal percentage of Cd(II) ion was increased with increase in pH from 2.0 to 4.0. This trend may be attributed to increased positive charge (protons) density on biosorbent surface at lower pH values (2.0–4.0) restricting the approach of metal cations towards biosorbent due to repulsive forces. In contrast, at higher pH values the biomass surface becomes negatively charged and the biosorption of the positive metal ions increased. A similar trend was observed for biosorption of cadmium onto olive pomace [32]. Thus, a maximum biosorption was observed at pH 5.0. Decrease in biosorption at higher pH could be due to the formation of soluble hydroxylated complexes of the cadmium ions, the concentration free cadmium ions decrease thereby at higher pH removal percentage decreased. Therefore, in present investigation optimum pH was chosen 5.0.

### 3.3. Effect of Biosorbent Dosage

Adsorbent dosage is an important parameter in adsorption studies because it determines the capacity of adsorbent for a given initial concentration. The effect of dose of biosorbent on the removal of metal ion was studied by increasing adsorbent dose from 50 mg to 1000 mg, keeping other parameters, that is, pH, initial concentration, and contact time constant. Maximum removal of metal ion was observed with a dosage of 300 mg of biosorbent and after that removal efficiency was decreased. This may be attributed to the fact that the number of active sites increased with dosage; hence, higher dose of adsorbent has positive effect on the initial rate of metal ion removal.

### 3.4. Adsorption Kinetic Studies

**Figure 5:**
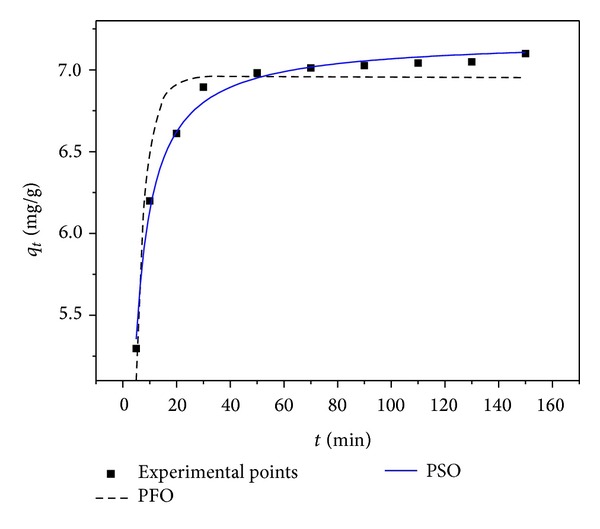
Pseudosecond-order kinetic model for Cd(II) onto WAS.

Sorption kinetics provides valuable insights into the reaction pathways and into the mechanism of sorption reactions. Several kinetic models are available to understand the behavior of biosorbent and also to examine the rate controlling mechanism of adsorption process. In order to analyze the biosorption kinetics of Cd(II) ions onto WAS, well-known kinetic models, Lagergren's pseudo-first-order, McKay, and Ho's pseudo-second-order and intraparticle diffusion models were applied to analyze the experimental data. The first-order rate equation of Lagergren [33] is one of the most widely used in the studies of sorption of a solute from liquid solution. It is represented as

(2) $$\begin{array}{c} q_{t}=q_{e}\left(1-e^{-k_{1}t}\right), \end{array}$$

where *q*_*e*_ (mg g^−1^) and *q*_*t*_ (mg g^−1^) are the adsorption amount at equilibrium and time *t* (min), respectively. *k*_1_ (min^−1^) is the rate constant in the pseudo-first-order (PFO) adsorption process. The pseudo-second-order (PSO) kinetic model, proposed by Ho and McKay [34], is based on the assumption that the adsorption follows second-order chemisorption. The non-linear form of PSO equation can be written as

(3) $$\begin{array}{c} q_{t}=q_{e}\frac{q_{e}k_{2}t}{1+q_{e}k_{2}t}, \end{array}$$

where *k*_2_ (g mg^−1^ min^−1^) is the rate constant of adsorption. The non-linear plots for both pseudo-first order and pseudo-second order model were shown in Figure 5. The values of *k*_2_ and *R* ^2^, along with the calculated uptake capacity *q*_*e*_, are provided in Table 2. Calculated correlation coefficients are close to unity for pseudo-second-order kinetic model; therefore, the sorption kinetics of Cd(II) could well be approximated more favorably by second-order kinetics model rather than pseudo-first-order kinetic model. The initial adsorption rate, *h*_0_ (mg g^−1^ min^−1^), is defined as

(4) $$\begin{array}{c} h_{0}=k_{2}q_{e}^{2}. \end{array}$$

**Figure 6:**
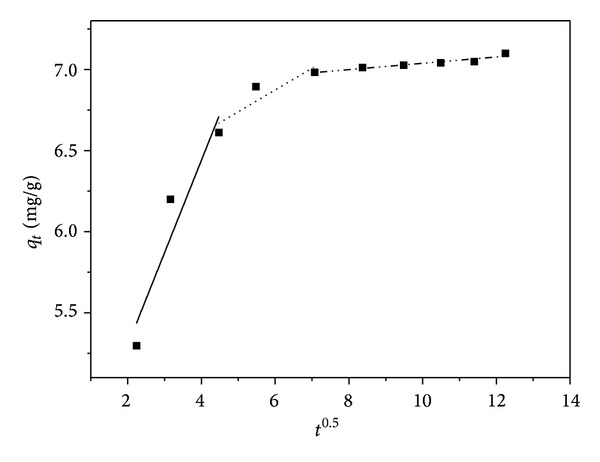
Intraparticle diffusion kinetics for adsorption of Cd(II) onto WAS.

During sorption process, there is a possibility of intra-particle pore diffusion of Cd(II) ions, which is often the rate-limiting step. The intra-particle diffusion becomes pertinent for batch mode of operation as diffusion varies with square root of time. Weber and Morris [35] correlated the same with time as:

(5) $$\begin{array}{c} q=K_{i}t^{1/2}, \end{array}$$

where *q* (mg g^−1^) is the adsorbed metal amount, *K*_*i*_ intraparticle diffusion rate constant (mg g^−1^ min^−1/2^). According to this model, the plot of uptake (*q*) versus the square root of time should be linear if intraparticle diffusion is involved in the adsorption process. If these plots pass through the origin then intraparticle diffusion is the rate-controlling step. In Figure 6, the intercept does not pass through the origin, which indicates that the pore diffusion is not the only rate-limiting step for the sorption of Cd(II) onto WAS. However, by comparing constants of all kinetic models, it can be concluded that the experimental data fit better to the pseudo-second-order kinetic model.

### 3.5. Analysis of Adsorption Isotherms

**Figure 7:**
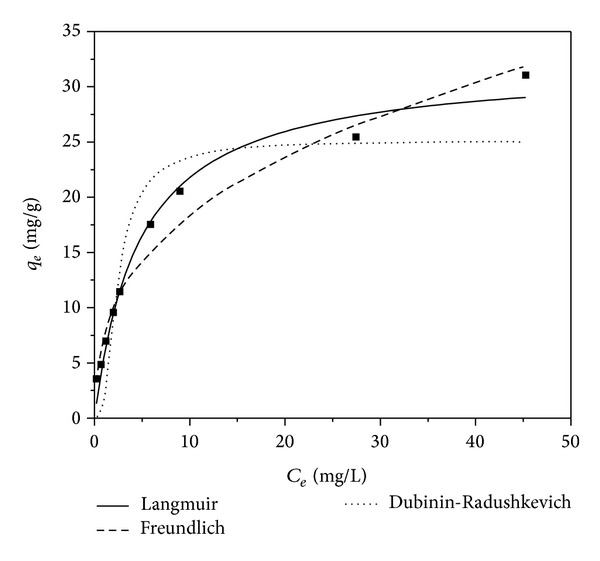
Biosorption isotherms of Cd(II) onto WAS.

Adsorption isotherm provides a relationship between concentration of metal ion in solution and the amount of the same adsorbed on the adsorbent when both the phases are in equilibrium. In the present study, the cadmium(II) uptake capacity of WAS was evaluated using the Langmuir, Freundlich, and Dubinin-Radushkevich (D-R) adsorption isotherm models and the plots are shown in Figure 7. The Langmuir equation can be written in the following form [36]:

(6) $$\begin{array}{c} q_{e}=\frac{q_{m}K_{L}c_{e}}{1+K_{L}c_{e}}, \end{array}$$

where parameters *q*_*m*_ and *b* are Langmuir constants related to maximum adsorption capacity (monolayer capacity) and bonding energy of adsorption, respectively, which are functions of the characteristics of the system as well as time. The Langmuir equation is used for homogeneous surfaces. The values of Langmuir parameters,*q*_max⁡_ and *b*, were 32.07 mg g^−1^ and 0.211 L mg^−1^, respectively. The correlation coefficient, *R* ^2^, was found to be 0.9812 (shown in Table 3). The essential features of the Langmuir biosorption isotherm can be expressed in terms of a dimensionless constant separation factor (RL), which is defined in (9):

(7) $$\begin{array}{c} R_{L}=\frac{1}{\left(1+K_{L}C_{0}\right)}, \end{array}$$

where *K*_*L*_ is the Langmuir constant (L mg^−1^) and *C*_0_ is the initial adsorbate concentration (mg L^−1^). The values of *R*_*L*_ in the range of 0-1 at all initial Cd(II) concentrations confirm the favorable uptake of cadmium process [15]. Freundlich isotherm is an empirical equation. This equation is one among the most widely used isotherms for the description of adsorption equilibrium. Freundlich isotherm is capable of describing the adsorption of organic and inorganic compounds on a wide variety of adsorbents including biosorbent [16]. This equation has the following form:

(8) $$\begin{array}{c} q_{e}=K_{F}C_{e}^{1/n}. \end{array}$$

Freundlich isotherm has the ability to fit nearly all experimental sorption-desorption data and is also applicable for fitting data from highly heterogeneous sorbent systems. Accordingly, this isotherm can adequately represent the sorption isotherm for most of the systems studied. The correlation coefficients (*R* ^2^), *K*_*F*_, and *n* were found to be 0.9682, 7.932, and 7.932, respectively. The magnitude of *K*_*F*_ and *n* shows easy separation of heavy metal ion from wastewater and high adsorption capacity. The 1/*n* values were between 0 and 1 indicating that the sorption of WAS was favorable at the studied conditions. Dubinin and Radushkevich conceived this equation for subcritical vapors in micropore solids where the adsorption process follows a pore filling mechanism onto energetically nonuniform surface [17]. The Dubinin-Radushkevich (D-R) model, which does not assume a homogeneous surface or a constant sorption potential as the Langmuir model, was also used to test the experimental data:

(9) $$\begin{array}{c} q_{e}=Q_{m}\exp\left(-K{\left[RT\ln\left(1+\frac{1}{C_{e}}\right)\right]}^{2}\right), \end{array}$$

(10) $$\begin{array}{c} q_{e}=Q_{m}\exp\left(-K\varepsilon^{2}\right), \end{array}$$

where *Q*_*m*_ is the maximum amount of the metal ion that could be sorbed onto unit weight of sorbent (mg g^−1^), *ε* is the Polanyi potential which is equal to *RT*ln⁡⁡(1 + 1/*C*_*e*_), where *R* and *T* are the universal gas constant (kJ mol^−1^ K^−1^) and the absolute temperature (*K*), respectively. The *K* in (9) and (10) is related to the mean free energy of sorption per mole of the sorbate when it is transferred to the surface of the solid from infinity in the solution and this energy can be computed using the following relationship:

(11) $$\begin{array}{c} E=\frac{1}{\sqrt{2K}}. \end{array}$$

The values of correlation coefficients (*R* ^2^) of all four adsorption models shown in Table 3 indicate that the Langmuir isotherm model exhibits a better fit to the equilibrium data than Freundlich and Dubinin-Radushkevich adsorption isotherms. Therefore, the biosorption process of Cd(II) by WAS can be interpreted as monolayer adsorption.

### 3.6. Desorption of Cd(II) from Sorbent

**Figure 8:**
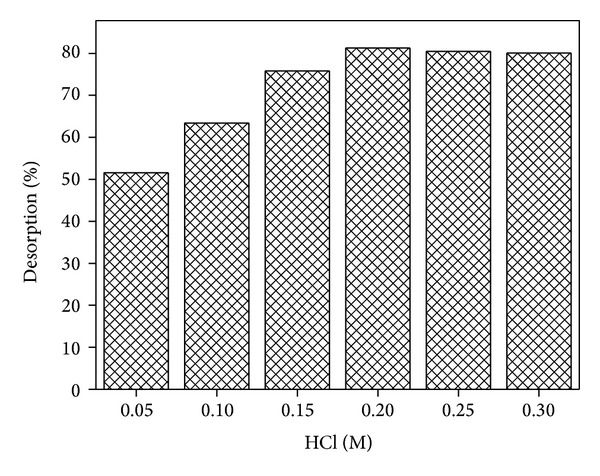
Desorption of Cd(II) ions from WAS.

Desorption studies were useful to identify the nature of biosorption process and to recover the metal from sorbent. Moreover, it also will help to regenerate the sorbents reuse to adsorb metal ions and to develop the successful sorption process. In present study, HCl was used as desorbing agent with various concentrations (0.05 M, 0.1 M, 0.15 M, 0.2 M, 0.25 M, and 0.3 M) and the results are shown in Figure 8. It can be clear from the results that the percent recovery of Cd(II) was increased with increasing the concentration of HCl from 0.05 M to 0.20 M and then remained almost constant.

### 3.7. Comparison with Other Biosorbents

A comparative data of Cd(II) sorption capacity of WAS with other biosorbents reported in the literature [18–24, 37–39] is presented in Table 4. The results showed that WAS has higher sorption capacity in removing Cd(II) from aqueous solutions compared to other biosorbents.

## 4. Conclusion

A new biosorbent was prepared by using wood apple shell (WAS) powder. Results obtained from the abovementioned systematic studies showed that maximum cadmium removal by adsorbent was observed at pH 5.0. Equilibrium adsorption showed that the biosorption process followed Langmuir adsorption isotherm model better than Freundlich, and D-R isotherm models, which indicates that monolayer adsorption capacity for Cd(II) was 32.071 with a correlation coefficient 0.98. The kinetics studies indicated that cadmium removal followed pseudo-second-order rate equation. Desorption studies showed that maximum recovery was achieved by 0.2 M HCl. This study demonstrated that WAS could be used as an effective biosorbent for removal of cadmium ions from wastewater.

## Figures and Tables

**Table 1:** Characteristics of wood apple shell (WAS) biosorbent.

Characteristics	Values
Bulk density (g/cm^3^)	0.52 ± 0.02
Moisture content (%)	5.91 ± 1.32
Ash content (%)	0.57 ± 0.01
Carbon (%)	47.66 ± 2.35
Hydrogen (%)	5.89 ± 0.03
Nitrogen (%)	0.10 ± 0.01
Oxygen^a^ (%)	45.58 ± 1.45
Electrical conductivity (µs/cm)	23.3 ± 1.23

^a^Estimated by difference.

**Table 2:** Pseudo-first-order and pseudo-second order rate constants for Cd(II) binding by WAS.

*q*_*e* exp⁡_ (mg g^−1)^	Pseudo-first-order model				Pseudo-Second-order model		
	*K*_1_ (min^−1^)	*q*_*e* cal_ (mg g^−1^)	*R* ^2^	*K*_2_ (mg^−1^ min^−1^)	*q*_*e* cal_ (mg g^−1^)	*h* (mg g^−1^ min^−1^)	*R* ^2^
7.099	0.267	6.953	0.9043	0.081	7.188	4.185	0.9917

**Table 3:** Isotherm parameters for Cd(II) biosorption by WAS.

Model	Values
Langmuir	
*Q*_*m*_ (mg g^−1^)	32.071
*b* (l mg^−1^)	0.211
*R* ^2^	0.9812
Freundlich	
*K*_*f*_ (mg g^−1^)	7.932
*n* (g L^−1^)	2.745
*R* ^2^	0.9682
Dubinin-Radushkevich	
*Q*_*m*_ (mg g^−1^)	25.058
*E*	8.838
*R* ^2^	0.8408

**Table 4:** Comparison of maximum adsorption capacities (*Q*_max⁡_) of Cd(II) with various biosorbents.

Biosorbent	*Q*_max⁡_ (mg g^−1^)	pH	Reference
Pine bark	28.0	7.5	[15]
Waste tea leaves	31.48	5.0	[16]
Hazelnut shells	5.42	6.0	[17]
Peat	22.5	5.0	[18]
Coffee husk	6.9	4	[19]
Heartwood powder of *Areca catechu *	10.66	6	[20]
Fennel biomass	26.59	4.3	[21]
Coconut copra meal	4.99	6.0	[22]
Papaya wood	17.22	5.0	[23]
Sugarcane bagasse	6.97	7.0	[24]
WAS	32.07	5.0	This study
